# Supplementary material for: Renal staffs’ understanding of patients’ experiences of transition from peritoneal dialysis to in-centre haemodialysis and their views on service improvement: A multi-site qualitative study in England and Australia
Source: PLoS One. 2021 Jul 19;16(7):e0254931. doi: 10.1371/journal.pone.0254931 (PMC8289060; doi:10.1371/journal.pone.0254931)
Supplement: S3 File — (DOCX) [file pone.0254931.s003.docx]

# S3 File: STAFF FOCUS GROUPS TOPIC GUIDE

Welcome and Introductions

We are talking with staff about how patients and caregivers experience changing treatments, from PD to haemodialysis. We are interested in hearing about what these changes in treatment mean to patients, caregivers and staff, how patients and caregivers cope, and how individual staff and renal units can support them through the change. This should help renal units to improve care and provide better support when treatments might need to be changed.

1) So can we start by everyone introducing themselves to the group. Can you tell us your name and a bit about your role?

Can you also say what involvement you have when patients change from PD to haemodialysis, or any other major treatment changes?

*(Explore: role of the service overall in supporting transitions; roles of particular individuals/professional groups)*

2) So let’s move on to thinking about how patients experience the transition from PD to HD:

- Could a couple of you tell us about the last patient you know of who made the transition, and what you saw happen to them?
- What roles did staff have to play in easing the transition?
- How typical are these examples?
- What so you think are the main barriers and difficulties to a smooth transition?
- And what makes it go well?

3) And what about the experiences of the caregivers?

4) What differences do you think there are for patients and caregivers between planned and unplanned transitions?

5) So do you think practice needs to change in renal units or not?

- If not, why not? If yes, how does practice need to change? And how easy/difficult do you think this will be and why?
- Overall, what do you think are the priorities for improving practice?
